# Diagnostic value of anti-cyclic citrullinated peptide antibodies in Greek patients with rheumatoid arthritis

**DOI:** 10.1186/1471-2474-8-37

**Published:** 2007-04-20

**Authors:** Ioannis Alexiou, Anastasios Germenis, Athanasios Ziogas, Katerina Theodoridou, Lazaros I Sakkas

**Affiliations:** 1 Department of Rheumatology, Thessaly University School of Medicine and Hospital, 412 22 Larisa, Greece; 2 Department of Immunology and Histocompatibility, Thessaly University School of Medicine and Hospital, Larisa, Greece

## Abstract

**Background:**

Anti-cyclic citrullinated peptide (anti-CCP) antibodies have been of diagnostic value in Northern European Caucasian patients with rheumatoid arthritis (RA). In these populations, anti-CCP antibodies are associated with the HLA-DRB1 shared epitope. We assessed the diagnostic value of anti-CCP antibodies in Greek patients with RA where the HLA shared epitope was reported in a minority of patients.

**Methods:**

Using an enzyme-linked immunosorbent assay (ELISA) (CCP2) kit, we tested anti-CCP antibodies in serum samples from 155 Greek patients with RA, 178 patients with other rheumatic diseases, and 100 blood donors. We also determined rheumatoid factor (RF) and compared it to anti-CCP antibodies for area under the curve (AUC), sensitivity, specificity and likelihood ratios.

**Results:**

Sensitivity of anti-CCP2 antibodies and RF for RA was 63.2% and 59.1%, and specificity was 95.0% and 91.2%, respectively. When considered simultaneously, the AUC for anti-CCP antibodies was 0.90 with 95% CI of 0.87 to 0.93 and the AUC for RF was 0.71 with 95% CI of 0.64 to 0.77. The presence of both antibodies increased specificity to 98.2%. Anti-CCP antibodies were positive in 34.9% of RF-negative RA patients. Anti-CCP antibodies showed a correlation with the radiographic joint damage. Anti-CCP-positive RA patients had increased the swollen joint count and serum CRP concentration compared to anti-CCP-negative RA patients (Mann-Whitney U test, p = 0.01, and p < 0.001, respectively). However, no correlation was found between anti-CCP antibodies and DAS28 score (r = 0.13, p = 0.12).

**Conclusion:**

In Greek patients with RA, anti-CCP2 antibodies exhibit a better diagnostic value than RF and a correlation with radiological joint damage and therefore are useful in everyday rheumatology practice.

## Background

In recent years it has become clear that early aggressive treatment in rheumatoid arthritis (RA) reduces joint damage and improves function [1]. To use a potentially toxic therapy as early as possible we require an accurate diagnosis of RA and also information about prognosis in an individual patient. Today, the diagnosis of RA depends mainly on clinical criteria that may take years to fulfill. Apart from clinical features, autoantibodies contribute to the diagnosis of various autoimmune diseases. In RA, rheumatoid factor (RF) has a fair sensitivity but a low specificity, since it is present in other rheumatic diseases, in infections, and in healthy people, especially the elderly [2].

In recent years, the introduction of serum antibodies against citrulline-containing molecules showed some promise as diagnostic tools in RA. Citrulline can be formed by posttranslational enzymatic conversion of arginine residues, catalyzed by peptidylarginine deiminase enzymes. Citrullinated molecules, the targets of these antibodies, include filaggrin, keratin, fibrin, and vimentin [3]. These antibodies are detected by ELISA, where a synthetic cyclic citrullinated peptide (CCP) is used as substrate. After the first generation of anti-CCP test (CCP1) [4,5] a second generation of anti-CCP test (CCP2) has been introduced. The sensitivity of anti-CCP2 test in various populations ranges between 64% and 74% whereas the specificity ranges between 90% and 99% [6-11]. A correlation of anti-CCP antibodies with radiographic joint damage has also been reported [12-15].

In Northern European Caucasian populations, RA has been found to be associated with the HLA-DRB1 shared epitope. In these populations, anti-CCP antibody production is associated with the HLA-DRB1 shared epitope [14]. However, the shared epitope has been detected in a minority of Greek patients with RA [16]. Therefore, we studied the diagnostic and prognostic value of anti-CCP antibodies in Greek patients with RA.

## Methods

### Patients

One hundred and fifty five Greek patients with RA (females 118, males 37; age 60.3 ± 12.8 years [mean ± SD]) attending the Rheumatology Department of Thessaly University Hospital, Larisa, were included in the study. All patients had RA according to American Rheumatism Association 1987 criteria [17]. In these patients age, sex, disease duration, clinical characteristics, basic blood and biochemistry tests, and medications were recorded. Forty four patients were on methotrexate, 37 patients were on leflunomide, 22 patients on hydroxychloroquine, 9 patients on prednizolon, 14 patients on cyclosporin-A, 4 patients on sulfasalazine, and 25 patients on anti-TNFα agents plus methotrexate.

One hundred and seventy eight patients with other diseases (females 130, males 48; age 54.9 ± 16.5 years) served as disease control. All these patients fulfilled the appropriate criteria for their specific disease. One hundred blood donors (females 15, males 85; age 39.2 ± 10.4) of the regional blood transfusion center, served as normal control. This study was approved by the Ethical Committee of our Institution.

### RA activity

Disease activity was assessed using the DAS28 score [18], morning stiffness (in minutes), extra-articular manifestations and C-reactive protein concentration (CRP; mg/dL).

### Radiographic assessment

An anteroposterior view X-rays of wrists and hands was obtained. Two rheumatologists (I.A, A.Z), used a Larsen's modification [19,20] score to assess radiographic joint damage in a 0 (no abnormality) to 5 (severe abnormality) scale, in a blind fashion regarding serology and clinical status. When needed, the two readers chose the lower score.

### Serology

Serum samples from patients and controls were kept at -80°C until tested. Anti-CCP antibodies were detected using the QUANTA lite CCP2 IgG ELISA kit (INOVA diagnostics, San Diego CA). The assay was performed according to the manufacturer's instructions (cut off value, 20 IU/mL). IgM RF was detected by immuno-nephelometry (Dade Behring, Marburg, Germany) (cut-off value, 15 IU/mL). Both assays were performed in blind fashion considering the final diagnosis and each other's result.

### Statistical analysis

Receiver operating characteristic (ROC) curves were drawn and the area under the curve (AUC) along with corresponding confidence intervals (CIs) was calculated. Diagnostic characteristics were determined by means of sensitivity, specificity, positive likelihood ratio and negative likelihood ratio and their CIs thereof with respect to the gold standard. Correlation between anti-CCP2 antibody levels and Larsen score was determined by the Spearman's rank correlation test. All reported p-values were two-tailed and statistical significance was considered at 0.05 level. Statistical analyses were carried out using the SPSS software.

## Results

### Frequency of anti-CCP2 antibodies

Demographic, clinical and laboratory features of RA patients are shown in Table 1. Frequencies of anti-CCP antibodies and RF in patients and controls are shown in Table 2. Among patients with RA, 98 patients (63.2%) had anti-CCP antibodies and 92 patients (59.1%) had RF. Seventy six RA patients (49.0%) were both anti-CCP-positive and RF-positive, 22 RA patients (14.2%) were anti-CCP-positive and RF-negative, and 16 RA patients (10.3%) were anti-CCP-negative and RF-positive. Anti-CCP and/or RF positivity was present in 75.5% of RA patients. Among 63 RF-negative RA patients, 22 patients (34.9%) were anti-CCP-positive. Among 57 anti-CCP-negative RA patients, 28.1% were RF-positive.

In our non-RA disease control group, 14 patients (7.9%) had anti-CCP antibodies. Nine of those 14 patients had very low antibody titres (23.2–27.7 IU/mL). One patient with systemic lupus erythematosus and a high anti-CCP antibody titre, had a mother with RA.

In the normal control group, none had anti-CCP antibodies and 3% had RF (Table 2).

### Diagnostic value of anti-CCP2 antibody test in rheumatoid arthritis

**Figure 1:**
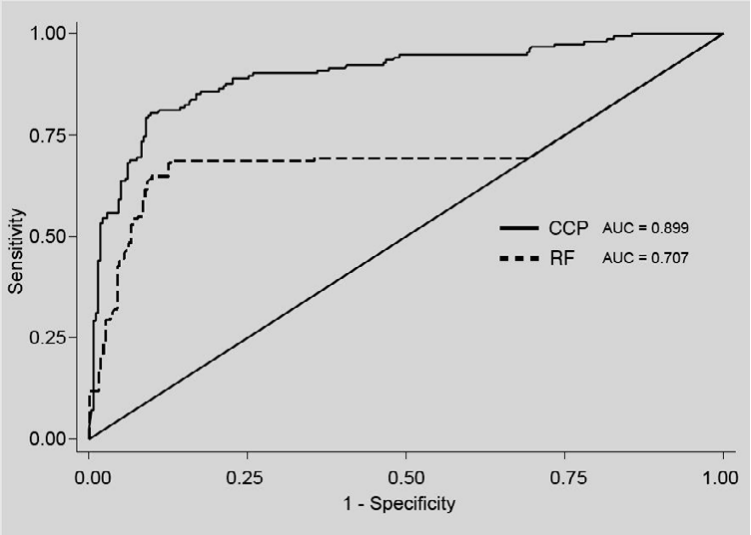
Receiver Operating Characteristic (ROC) curve of anti-CCP2 antibodies and rheumatoid factor.

For anti-CCP antibodies and RF, diagnostic value was described by ROC (receiver operating characteristic) curve, AUC (Figure 1), sensitivity and specificity, positive and negative predictive values (Table 3). When sensitivity and specificity were considered simultaneously, the AUC for anti-CCP antibodies was 0.90 with 95% CI of 0.87 to 0.93, whereas the AUC for RF was 0.71 with 95% CI of 0.64 to 0.77. Therefore, anti-CCP antibodies exhibited a better diagnostic value than RF in Greek patients with RA. When sensitivity and specificity were considered independently, sensitivity of anti-CCP antibody test for RA was 63.2% and specificity was 95.0%, whereas the respective values for RF were 59.1% and 91.2%.

The presence of either antibody (anti-CCP antibody and/or RF) increased sensitivity (75.5%), whereas the presence of both antibodies (anti-CCP antibody and RF) increased specificity (98.2%).

### Anti-CCP2 antibodies and RA activity indices

Disease activity, as defined by the DAS28 score, was low (DAS28 < 3.2) in 30 RA patients (19.7%), moderate (DAS28 3.2–5.1) in 68 RA patients (44.7%), and high (DAS28 > 5.1) in 54 RA patients (35.5%). RA patients with high DAS28 score had a higher frequency of anti-CCP antibodies (75.9%) compared to RA patients with low DAS28 score (31.8%) (p < 0.001). However, no correlation was found between anti-CCP antibodies and disease activity as defined by the DAS28 score (r = 0.13, p = 0.12). Anti-CCP-positive RA patients had increased swollen joint count and serum CRP concentration compared to anti-CCP-negative RA patients (Mann-Whitney U test, p = 0.01, and p < 0.001, respectively).

Thirty nine RA patients (72.2%) with high disease activity were RF(+), compared to 15 RA patients (50.0%) with low disease activity (not significant). Serum IgM RF levels showed a correlation with DAS28 score (r = 0.29, p = 0.001). Also, RF(+) RA patients had increased swollen joint count and serum CRP concentration compared to RF(-) RA patients (Mann-Whitney U test, p = 0.02, and p = 0.002, respectively).

### Anti-CCP2 antibodies and radiographic joint score

Twelve RA patients had Larsen radiographic score 0-I, 83 patients had score II-III and 36 patients had score IV-V. Among RA patients with Larsen score IV-V, 28 patients (77.7%) were anti-CCP-positive and 22 patients (61.0%) were both anti-CCP-positive and RF-positive. The respective numbers among RA patients with the Larsen score 0–1 were 6 (50.0%) and 3 (25.0%). Patients with anti-CCP antibodies had a trend towards a more severe radiographic joint damage, compared to patients without anti-CCP antibodies, but this trend did not reach statistical significance. However, there was, although a fairly weak, correlation between radiographic joint score and anti-CCP antibodies (Spearman correlation coefficient, r = 0.27, p = 0.001). When RA patients were divided according to disease duration (0–5 years, 5–10 years, more than 10 years), a correlation of radiographic score with anti-CCP antibodies was found in the group with short disease duration (0–5 years) (Spearman r = 0.40, p = 0.029). Also, a correlation was found between RF and radiographic joint score (Spearman r = 0.33, p= 0.001).

## Discussion

The first study on anti-CCP antibodies (CCP1 test) reported a sensitivity of 68% and specificity of 98% for RA [4], whereas the same investigators when analyzed patients from other centers found sensitivity 45–80% and specificity 96–100% [4]. Other studies utilized CCP2 kits found sensitivity 64–74% and confirmed the high specificity of the test (90–99%) [6-12]. Anti-CCP antibodies (CCP1 test) have been used successfully to diagnose persisted arthritis as compared to self-limited arthritis [5], or to differentiate RA (CCP2 test) from early undifferentiated arthritis [21]. Our findings of sensitivity of 63.2% and specificity of 95% for anti-CCP antibodies in RA are in agreement with these studies. The different frequencies of anti-CCP antibodies in various RA patient cohorts can be explained as follows: anti-CCP antibodies are directed against different epitopes in citrulline-containing molecules and sera from individual patients may contain different subsets of anti-CCP antibodies [22,23]. As observed with other autoantibodies in autoimmune diseases, the production of anti-CCP antibodies in RA is influenced by HLA alleles. Serum anti-CCP antibody levels were higher in RA patients with the shared HLA-DRB1 (SE) epitope than in RA patients lacking the SE epitope [14]. Finally, RA treatment may decrease serum anti-CCP antibody levels. Anti-TNFα treatment has been found to decrease serum anti-CCP antibody levels [24]. The high specificity of anti-CCP antibodies is particularly useful in RF-negative RA patients. In our study, the frequency of anti-CCP antibodies in RF-negative RA patients was 34.9%, but this has been reported up to 40% [9].

Nearly a third of anti-CCP-negative RA patients (28.1%) were RF-positive. The relatively low percentage of RF in our RA group may be explained by the fact that our RA patients encompass the full range of RA severity/activity because of the Health system in Greece: Our Hospital functions as a primary as well as secondary and tertiary center. In other countries, where University Hospitals may only accept referrals from general practitioners, RA patients may include the more severe cases.

In agreement with another study [9], our study found an association of anti-CCP antibodies with disease activity as defined by swollen joint count and increased CRP concentration. Our study is a cross-sectional study and has not followed RA patients over years to address the significance of anti-CCP antibodies in joint damage. Nevertheless, our study demonstrated a correlation between anti-CCP antibodies and radiological joint score in patients with RA. Similarly, anti-CCP antibodies alone or together with the HLA-DRB1 shared epitope have been found to be associated with radiological joint damage [12]. In longitudinal studies, anti-CCP antibodies alone [15] or together with the HLA-DRB1 shared epitope [14] have been associated with faster radiological joint progression. In multiple regression analysis, anti-CCP1-positivity [25] and anti-CCP2-positivity [13] have been found to be predictor of radiological joint outcome. Taken together these findings suggest that anti-CCP antibodies are useful in predicting a severe disease course in RA.

The moderate sensitivity and high specificity of anti-CCP antibodies for RA, along with the appearance of anti-CCP antibodies before disease onset [26,27], suggest that anti-CCP antibodies be included in the classification criteria for RA. Furthermore, the correlation of anti-CCP antibodies with radiographic joint damage in RA [13-15,25] provides additional aid for clinicians in deciding early aggressive treatment for a RA patient who is likely to have a severe disease course.

## Conclusion

Anti-CCP2 antibodies had a better diagnostic value than RF for RA in Greeks. A considerable proportion (34.9%) of RF-negative RA patients were anti-CCP-positive. Furthermore, RA patients with anti-CCP antibodies were more likely to have more radiographic joint damage than patients without anti-CCP antibodies. These results confirm results in other populations and suggest that anti-CCP2 test is useful in everyday rheumatology practice.

## Competing interests

The author(s) declare that they have no competing interests.

## Authors' contributions

IA: examination of patients, collection of data, reviewing of x-rays, analysis of data, writing of manuscript.

AG: concept, testing for anti-CCP antibodies and RF, reviewing of manuscript.

AZ: examination of patients, reviewing of x-rays

KT: testing for anti-CCP antibodies and RF

LIS: concept, examination of patients, reviewing of manuscript

All authors have read and approved the final manuscript

## Pre-publication history

The pre-publication history for this paper can be accessed here:



## Figures and Tables

**Table 1:** Demographic and clinical characteristics of patients with rheumatoid arthritis

Feature	Value(mean ± SD)
Sex (%women)	76%
Age	60.3 (± 12.8) years
Disease duration (n = 153)	11.3 (± 0.8) years
Morning stiffness (n = 137)	32.9 (± 52.6) minutes
Tender joints (n = 153)	8.0 (± 7.0)
Swollen joints (n = 153)	3.0 (± 5.0)
ESR (n = 152)	30.9 (± 22.8) mm/1^st ^hour
CRP (n = 143)	1.8 (± 3.3) mg/dL
DAS28 (n = 152)	4.5 (± 1.6)
Larsen score (n = 131)	2.1 (± 1.1)
Anti-CCP antibodies (n = 155)	81.9 (± 115.4) IU/mL
RF (n = 155)	246.3 (± 508.1) IU/mL

**Table 2:** Anti-CCP2 antibodies and RF in RA and non-RA patients and normal controls.

Disease (n)	anti-CCP (%)	RF (%)
Rheumatoid arthritis (155)	98 (63.2)	92 (59,1)
Spondyloarthropathies (53)	2 (3.8)	5 (9.4)
Systemic lupus erythematosus (32)	2 (6.2)	6 (18.7)
Sjogren's syndrome (17)	3 (17.6)	3 (17.6)
Osteoarthritis (19)	0 (0.0)	0 (0.0)
Vasculitis (15)	3 (20)	4 (26.7)
Polymyalgia rheumatica (11)	0 (0.0)	0 (0.0)
Crystal arthritis (7)	0 (0.0)	1 (14.3)
Adult onset Still's disease (5)	0 (0.0)	0 (0.0)
Systemic sclerosis (5)	1 (20)	1 (20)
Juvenile idiopathic arthritis (4)	1 (25)	1 (25)
Brucellosis (4)	2 (50)	1 (25)
Others (n = 6)	0 (0.0)	0 (0.0)
Normal controls (100)	0 (0.0)	3 (3.0)

Others = mixed connective tissue disease, familial Mediterranean fever, rheumatic fever, dermatomyositis, anti-phospholipid syndrome.

**Table 3:** Diagnostic performance of anti-CCP2 antibodies and RF in rheumatoid arthritis

**Anti-CCP2**	R.A. vs non R.A.	R.A. vs other diseases	R.A. vs blood donors
AUC	0.90 (95% CI: 0.87 – 0.93)	0.87 (95% CI: 0.83 – 0.91)	0.95 (95% CI: 0.92 – 0.97)
Sensitivity*	63.6% (95% CI: 55.8% – 70.8%)	63.6% (95% CI: 55.8% – 70.8%)	63.6% (95% CI: 55.8% – 70.8%)
Specificity*	95.0% (95% CI: 91.7% – 97.0%)	92.1% (95% CI: 87.2% – 95.3%)	100% (95% CI: 96.4% – 100%)
PPV*	87.5% (95% CI: 80.1% – 92.4%)	87.5% (95% CI: 80.1% – 92.4%)	100% (95% CI: 96.2% – 100%)
NPV*	83.8% (95% CI: 80.0% – 87.0%)	74.55% (95% CI: 68.4% – 79.8%)	64.1% (95% CI: 56.3% – 71.2%)
LR +*	12.6 (95% CI: 10.9 – 14.7)	8.1 (95% CI: 6.95 – 9.41)	Undefined
LR -*	0.38 (95% CI: 0.37 – 0.40)	0.39 (95% CI: 0.38 – 0.41)	0.36 (95% CI: 0.35 – 0.38)
Diagnostic OR*	33 (95% CI: 17.6 – 61.9)	20.5 (95% CI: 10.8 – 38.8)	Undefined

**RF**	R.A. vs non R.A.	R.A. vs other diseases	R.A. vs blood donors

AUC	0.71 (95% CI: 0.64 – 0.77)	0.719 (95% CI: 0.66 – 0.78)	0.682 (95% CI: 0.61 – 0.76)
Sensitivity*	59.09% (95% CI: 51.2% – 66.5%)	59.09% (95% CI: 51.2% – 66.5%)	59.09% (95% CI: 51.2% – 66.5%)
Specificity*	91.24% (95% CI: 87.3% – 94.0%)	87.93% (95% CI: 82.3% – 92.0%)	97.00% (95% CI: 91.6% – 99.0%)
PPV*	79.13% (95% CI: 70.8% – 85.6%)	81.25% (95% CI: 73.0% – 87.4%)	96.81% (95% CI: 91.0% – 98.9%)
NPV*	79.87% (95% CI: 75.1% – 83.9%)	70.83% (95% CI: 64.5% – 76.5%)	60.62% (95% CI: 52.9% – 67.9%)
LR +*	6.75 (95% CI: 6.13 – 7.43)	4.89 (95% CI: 4.39 – 5.46)	19.70 (95% CI: 10.10 – 38.43)
LR -*	0.45 (95% CI: 0.43 – 0.46)	0.47 (95% CI: 0.45 – 0.48)	0.42 (95% CI: 0.41 – 0.44)
Diagnostic OR*	15.05 (95% CI: 8.88 – 25.51)	10.52 (95% CI: 6.02 – 18.38)	46.70 (95% CI: 14.17 – 154.00)

The AUC for anti-CCP2 antibodies of 0.90 (95% CI, 0.87–0.93) is higher than the AUC for RF of 0.71 (95% CI, 0.64–0.77), with no overlapping of the two CIs, and shows that the diagnostic value of anti-CCP2 antibodies is higher than RF in rheumatoid arthritis.

AUC = area under the curve; PPV = positive predictive value; NPV = negative predictive value; LR+ = likelihood ratioof a positive test; LR- = likelihood ratioof a negative test OR = odds ratio
